# A comparative study of thermally and chemically treated dairy waste: Impacts on soil phosphorus turnover and availability using 33P isotope dilution

**DOI:** 10.1016/j.jenvman.2022.116702

**Published:** 2023-01-15

**Authors:** Olha Khomenko, Owen Fenton, J.J. Leahy, Karen Daly

**Affiliations:** a Teagasc, Johnstown Castle, Environment Research Centre, Wexford, Y35 TC97, Ireland; b Department of Chemical Sciences, School of Natural Sciences, University of Limerick, Limerick, V94 T9PX, Ireland

**Keywords:** Recycled dairy waste, Bio-based fertilizers, Phosphorus availability, Thermally treated, Isotope pool dilution, STRUBIAS

## Abstract

Dairy processing sludge (DPS) and DPS-derived secondary products such as struvite, biochar, hydrochar and ash (collectively known as SRUBIAS) are emerging as alternatives to fertilizers produced from mined rock phosphate. However, little is known about how these products affect soil P availability and daily P turnover rates.. A lack of such information prevents precision nutrient management planning using these products out on farms. This study used a novel isotope dilution technique (IPD) with ^33^P as a tracer to compare P turnover in soils amended with chemically (alum-treated DPS and struvite) and thermally (biochar, hydrochar, ash) treated DPS. Results showed that thermally treated products exhibited poor agronomic performance as P fertilizers, potentially inhibiting P availability when applied to soils. For example, a P deficient soil amended with hydrochar treatment at the highest application rates did not record a build-up of available P to agronomic target values. In ash and biochar treated P deficient soils, available P increased but only with very high application rates of 150 and 80 mg P kg ^−1^. The application of these products as fertilizers could have negative implications for both environmental and agronomic goals. Conversely, chemically treated fertilisers demonstrated better agronomic performance. The same agronomic target value was reached with application rates of only 20 mg P kg ^−1^ soil for DPS and 50 mg P kg ^−1^ soil for struvite. However, the techniques deployed revealed that these products exhibited slower rates of available and exchangeable P build-up when compared with chemical fertilisers. This suggests that these bio-based alternatives require higher application rates or earlier application times compared to conventional chemical fertilizers. Regulations providing advice on P use in agricultural soils need to account for slower P turnover in soils receiving recycled fertilizers. The IPD technique is transferrable to all wastes to examine their performance as fertilizers.

## Highlights

• Dairy waste (DPS), struvite, biochar, hydrochar, ash compared as P fertiliser.
• All products, except hydrochar increased P availability for grass growth.
• Low doses of ash, biochar and hydrochar decreased exchangeable P in soil.
• Whereas Struvite and DPS increased exchangeable P even at low application rates.
• At low application rate thermally treated products immobilized available P.

## Graphical abstract

**Figure:**
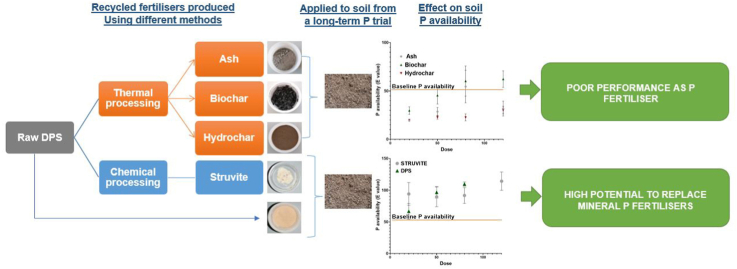

Abbreviations; Al Aluminium; Ca Calcium; DPS Dairy processing sludge; E Exchangeable phosphorus; Fe Iron; IEK Isotope exchange kinetics; IPD Isotope pool dilution; LR Lime requirement; M3 Mehlich3; Mg Magnesium; P Phosphorus; SSP Single superphosphate; TC Total carbon; TN Total nitrogen; TOC Total organic carbon; TP Total phosphorus; WHC Water holding capacity.

## 1 Introduction

In the European Union (EU) the introduction of the EU Circular Economy Package (EC, 2016) provides for the sustainable use of resources including the development of efficient and environmentally sustainable technologies. For agriculture, the introduction of the new course of development means an increased interest in returning recycled nutrients to the soil as fertilisers, thereby resolving the need for waste management and soil fertilisation. One of the soil nutrients included in the list of EU critical materials is phosphorus (P) (EC, 2014) due to its supply risk and importance as a resource. The Agricultural sector in the EU is highly dependent on imported P sources with less than 20% of P being mined in the EU (EC, 2017). The alternative P sources such as recycled food waste, animal manure and sewage sludge are being explored for use in agriculture as P fertilisers. Secondary fertilisers derived from raw waste were recently included in the revised EU fertiliser regulation (EC, 2019) supporting the transition to a circular economy. To accelerate the transition to sustainable agricultural systems the Farm to Fork Strategy under the EU Green Deal (EC, 2020) targets a reduction of fertiliser use by 20% and recommends the use of recycled organic wastes as a source of nutrients in soils.

Recycling agricultural wastes could provide an alternative source of P, and reduce the reliance on rock phosphate. Returning decomposed plant debris and animal waste to soils could provide up to 50% of the total P demand (Stutter et al., 2012). While some of the wastes such as animal manures and composted food waste have been used as fertilisers for decades, the potential for food production wastes is often overlooked as they are not widely recycled. The dairy industry alone can generate up to 20 kg of nutrient-rich dairy processing sludge (DPS) per cubic meter of processed milk (Ashekuzzaman et al., 2019) which can be used as a secondary material for P fertilisers production. In pasture-based systems, returning DPS to the soil would allow a transition to more sustainable nutrient management by returning P removed in products back to the soil. Both raw DPS and DPS-derived STRUBIAS products (STRUvite, BIochar, AShes) are being land applied as soil fertiliser with high nitrogen (N) and P content (Watkins and Nash, 2010; Cameron et al., 2002). However, their impact on soil P availability and cycling in soils is understudied. Previous studies on chemically precipitated DPS reported high variability in the availability of nutrients in the products depending on the wastewater treatment process (Ashekuzzaman et al., 2021a, Ashekuzzaman et al., 2021b). Currently, application rates for recycled fertilisers are based on the “Codes of Good Practice for the Use of Biosolids in Agriculture” (Fehily Timoney and Company, 1999) and are determined by the soil's nutrients demand, pH, and metals content, and on the recycled fertilisers' characteristics such as nutrients and contaminants content. Applying these recommendations to bio-based fertilisers can be problematic since the P availability, release dynamics and use efficiency in the recycled fertilisers is not equivalent to the conventional mineral fertilisers. This is linked to structural and chemical composition differences (Vanden et al., 2021; Khiari et al., 2020). Only a small fraction of soluble P added to the soil with fertiliser remains in the available P pool after crop uptake, the rest is distributed between less available P pools for later release into soil solution through desorption and dissolution processes (Sims and Baker, 2004). Previous studies reported that in recycled fertilisers the fraction of soluble P can be small, especially when the products contain P precipitating metals or undergo thermal treatment (Nanzer et al., 2014) which leads to the formation of stable P forms with limited availability for crops. Such products, may require higher application rates to meet crop demand, compared to immediately soluble mineral fertilisers. Khomenko et al. (submitted) reported that recycled dairy waste contained P in labile form that can be released over time, and application times need to be adjusted to account for the slower P release. . To provide the growers with an appropriate advice for recycled products, information on available soil P turnover rates is essential to amend current fertiliser recommendations.

In a previous study Khomenko et al. (submitted) a combination of extractive soil tests (Morgan's P and Mehlich3 P) and isotope pool dilution techniques were used to access the P availability and build-up dynamics in soils under long-term P management. These methods allow quantification of both orthophosphate ions in soil solution (available P), which reflects the amount of immediately available P, and the rapidly exchangeable orthophosphate ions (exchangeable P) which can replenish the solution P as it is being removed from the system by plants. The latest modifications of isotope tracing methods, the isotope pool dilution (IPD) technique (Di et al., 2000; Kellogg et al., 2006; Wanek et al., 2019), based on the nutrient transformation in soil model (Kirkham and Bartholomew, 1955) was used to quantify the daily rate of P transfer between the available P and the exchangeable P pools. The method can quantify the total daily P influx, which reflects the amount of P transferred from the P reserves into the available P pool in one day, and the total daily efflux reflecting the amount of P transferred back from the available into the exchangeable P pool (Wanek et al., 2019). The total P flux is a sum of biotic and abiotic processes and reflects both physiochemical and biological transformation of P in the studied system. The model allows for the quantification of the P fluxes in a system without isolating the available and exchangeable P pool from the system and the possibility of either P flux being significantly higher is taken into account (Kirkham and Bartholomew, 1955). The changes in the available and exchangeable P pools and rates of P turnover following the application of the novel recycled fertilisers are not well documented, however, this information is essential for management recommendations and advice on soil P build-up mechanisms using recycled P fertilisers. The objective of this study was to (1) compare chemically and thermally treated recycled DPS as fertilisers for the build-up of available and exchangeable soil P pools and (2) to describe mechanisms controlling P turnover and availability in soils receiving chemically and thermally treated recycled fertilisers.

This study used P derived from raw DPS and STRUBIAS to evaluate their impact on the P turnover mechanism in soils with varying P status. To meet the objective of the study the changes in the soil test P induced by the application of the recycled fertilisers were measured. The ability of the recycled fertilisers to build up soil P reserves, expressed as rapidly exchangeable P was examined. The P turnover rates were measured using a^33^P isotope dilution technique to assist in understanding of P build-up mechanism. The available and exchangeable P build-up rate and the P turnover rates were measured as a function of the type of the product and application rate. Single superphosphate (SSP) amendment was used as a reference treatment to compare the effect of the recycled and conventional mineral fertilisers on soil P availability and dynamics. Phosphorus turnover rates in soils amended with recycled and conventional fertilisers were compared and the predominant processes and direction of P flux governing soil P availability were identified.

## 2 Materials and methods

### 2.1 Soil sample collection and characterisation

A poorly-drained Gley soil from a long-term P trial established in 1968 at Johnstown Castle, County Wexford, Ireland was used in the study (Kurz et al., 2005; Tunney et al., 2010). Since the start of the trial, the soils have been receiving P fertilisers at different application rates (0, 15, and 30 kg P ha^−1^) resulting in plots with P inputs lower than P offtakes (P deficient), plots where P inputs are matching P offtakes (P balanced), and plots receiving P in excess of crop demand (P saturated). Three plots representing P deficient, P balanced, and P saturated soils were sampled in April 2020. A composite sample of the top 10 cm of soil was sampled from each plot. Soils were air-dried, homogenised and hand sieved to 2 mm. Three intact cores of 10 cm height and diameter were also collected from each paddock for the determination of soil bulk density using British standards methods (BS 1377-2, 1990). Water-holding capacity (WHC) was determined after full saturation followed by 48 h of free drainage (Achat et al., 2014). The plant available P was determined using Morgan's P test (Morgan, 1941). Mehlich3 extractable P, Al, Ca, Fe, and Mg were determined using in Mehlich3 method (Mehlich, 1984). The basic properties of these are summarized in Table 1. In Ireland, Morgan's P values are placed into bands or indices to indicate likely response to fertiliser for grassland production and easy management of soil P levels. The P Index system is outlined in Table 2 and soils in this study fell into P indices 1, 2 and 4 based on their long-term P management. According to the soil index system (Table 2), the deficient soil fell into P index 1. Typically soils in Index 1 are required to transition to index 3 in order to build up the available P pool to the agronomically optimal level. The P balanced soil was in the upper band of P index 2, and the P saturated was classified as P index 4 soil which requires P drawdown.

**Table 1:** The basic properties of the soils under long-term P management – total Phosphorus (TP) content, total carbon (TC), total nitrogen (TN) (Griffiths et al., 2012), pH, and bulk density.

	TP mg kg^−1^	TC, %	pH	Bulk density, g ml-1	
P deficient	408	4.1	5.45 5.66 5.62		0.90 0.87 0.84
P balanced	662	5.2			
P saturated	918	4.7

**Table 2:** The Soil Phosphorus Index system used for classification of grassland soils in Ireland (adapted from Wall and Plunkett, 2020).

Soil P index	Soil P range (Morgan's P mg L^−1^)	Index description	Response to fertilizer
1	0–3	Very low	Definite
2	3.1–5	Low	Likely
3	5.1–8	Medium/adequate	Unlikely
4	Above 8	Sufficient/High	None

### 2.2 Recycled dairy processing waste products and characterisation

Five recycled DPS fertilisers were examined in the current study, namely: bio-chemically treated activated dairy processing sludge (DPS) and second-generation products, ash, biochar, hydrochar, and struvite. The DPS was homogenised, and freeze-dried for 48 h (ScanVac, CoolSafe 55-9 Pro). The freeze-dried DPS, hydrochar, and biochar were ground and sieved to 0.2 mm to allow homogeneous distribution in the soils. The struvite was produced in a stirred batch reactor at 22 °C with the reaction time of 1 h and stirring rate of 60 rpm (Numviyimana et al., 2020). The biochar was produced from a mixture of DPS and spruce wood chips at a pilot-scale facility described by Kwapinska et al. (2019). The hydrochar was produced from Iron-treated DPS by placing the waste material was placed into the reactor liner with no additional water at 200 °C. The time required for the reactor to reach the set temperature was 3 h and the reaction time was 2 h. During the reaction the rector stirrer was operating at 25 rpm. After this the solid HC separated from the liquid portion through filtration and dried in an oven at 105 °C for 24 h (Shi et al., 2022). The ash was produced in a laboratory furnace at 650 °C from the biochar (Shi et al., 2022).

Organic matter (OM) and dry matter content (DM) were determined in the fertilisers using the standard gravimetric method 2540 G (APHA, 2005). The concentration of nutrients (P, K, and Ca), and metals (Al, Fe) were determined by an Agilent 5100 synchronous vertical dual view inductively coupled plasma optical emission spectrometer (Agilent 5100 ICP-OES) following microwave-assisted acid digestion of the samples. The samples were also analysed for total carbon (TC) and total nitrogen (TN) by the high temperature combustion method (LECO TruSpec CN analyser). Table 3 shows the results of the recycled DPS fertilisers’ analysis.

**Table 3:** Main characteristics of the recycled DPS fertilisers – pH, total P, N, Al, Ca, Fe, and C content.

Product	pH	TP, mg kg^−1^	TN, mg kg^−1^	Al, mg kg^−1^	Ca, mg kg^−1^	Fe, mg kg^−1^	TC,%
DPS	7.7	39,720	71,600	192,000	31,786	685	36.2
Ash	9.33	99,319	1100	82,090	227,522	7532	0.9
Biochar	N/A	52,292	19,400	33,776	97,035	4051	28.4
Hydrochar	6.9	78,856	37,500	7958	67,989	177,286	22.6
Struvite	N/A	104,215	10.7	20.17	14,657	73	10.7

### 2.3 Soil treatments and isotope pool dilution assay procedure

The IPD assay based on Kirkham and Bartholomew (1955) model of nutrient transformation in soil was used to determine daily total flux rates (Kruse et al., 2015; Wanek et al., 2019). Wanek et al. (2019) have successfully modified and applied this method to measure P turnover and optimised the conditions of the IPD assay to minimise error associated with the extraction methods sensitivity and quantification of P in the soil extracts. In the present study the two main P fluxes are determined in the soils: (1) total P influx rate representing the sum of daily biotic and abiotic fluxes of soil P into the available P pool and (2) total P efflux rate representing the reverse P flux from the available P pool back into the soil matrix. Unlike the traditional isotope exchange kinetics (IEK) approach this model is based on the equation linking time-resolved changes in the P concentration and specific activity in the soil solution over time. When paired with rapid and sensitive P extraction protocols the method allows for a more accurate determination of soil P flux rates, and provides information on both P influx into the available P pool and the reverse flux whilst the IEK method only provides information on P release (P mineralization rate) (Bünemann, 2015).

The P flux rates were measured in soil following incubation of soils with the recycled fertilisers. 100 g of air-dried and sieved soils were weighed into individual containers and amended with DPS, ash, biochar, hydrochar, and struvite at the following application rates: 20, 50, 80, 120, 150, and 180 mg P kg^−1^ soil. Soils which received 50 mg P kg^−1^ in form of single superphosphate (SSP) were used as a reference treatment. The products were incorporated into the soils, and soils were brought to 60% WHC and pre-incubated for 6 days at 37 °C. After the pre-incubation stage six aliquots were subsampled from each of the containers for the Isotope pool dilution (IPD) assay.

Each aliquot of the pre-incubated soils was labelled with 22,2 KBq of [P33]-Phosphoric acid >3000 Ci/mmol 10 mCi (370 MBq)/ml) ^33^P in the form of [^33^P]-Phosphoric acid (Hartmann Analytic GmbH) and thoroughly homogenised. Three replicates of each treatment were allowed to incubate for 4 h and the remaining replicates were incubated for 24 h. At the end of the incubation, soils were extracted using Morgan's reagent. The P concentration in soil extracts was measured colourimetrically using the molybdenum blue method (Pansu and Gautheyrou, 2006) using SPECORD 200 PLUS double-beam spectrophotometer (Analytik Jena GmbH, Germany). The Activity of the extracts was measured after mixing 0.5 ml of the soil extracts with 4 ml of scintillation cocktail (Ultima Gold, PerkinElmer) using a liquid scintillation counter Tri-Carb 5110 (PerkinElmer, United States). Due to the short half-life of the ^33^P all the activity measurements were decay-corrected using QuantaSmart software in-built tool.

### 2.4 Calculations of total rates of P turnover and isotopically exchangeable P

The calculations of the total P turnover rates were based on the following equation (Kirkham and Bartholomew, 1955):

(1) $$Cp-{Cp}_{0}=\left(I-Ef\right)\left(t-t_{0}\right)$$

where Cp – P concentration in soil solution after 24 h of incubation, mg L^−1^

Cp_0_ – P concentration in soil solution after 4 h of incubation, mg L^−1^

I – total P influx, mg P L^−1^ soil d^−1^

Ef – total P efflux, mg P L^−1^ soil d^−1^

t_0_ and t – incubation time 4 h and 24 h respectively, in days.

Depending on whether the influx (I) or the efflux (Ef) rate is a predominant flux, or the fluxes are equal, one of the following equations can be used (Kellogg et al., 2006):

(2) $$I>EfI=\frac{\left(cp-cp_{0}\right)}{\left(t-t_{0}\right)}\times \left(\frac{1n\left(\frac{SA_{t0}}{SA_{t}}\right)}{1n\left(\frac{Cp_{0}}{CP}\right)}\right)$$

(3) $$I<EfI=\frac{\left(cp-cp_{0}\right)}{\left(t-t_{0}\right)}\times \left(\frac{1n\left(\frac{SA_{t0}}{SA_{t}}\right)}{1n\left(\frac{Cp}{CP_{0}}\right)}\right)$$

(4) $$I=EfI=\frac{\left(cp-cp_{0}\right)}{\left(t-t_{0}\right)}\times \left(\frac{1n\left(\frac{SA_{t0}}{SA_{t}}\right)}{1n\left(\frac{Cp_{0}}{CP}\right)}\right)I=\frac{\left(cp-cp_{0}\right)}{\left(t-t_{0}\right)}\times ln\left(\frac{CP}{CP_{0}}\right)$$

where ${SA}_{t_{0}}$ and ${SA}_{t}$ is specific activity of the soil solution at 4 and 24 respectively, Bq L^−1^.

Knowing the value of total P influx, total P efflux can be calculated from equation (1).

In the isotopic dilution method applied in the current study a carrier-free isotope in the form of ^33^P phosphate was applied. Since in the labelled soil ^33^P and ^31^P phosphates have the same kinetic properties and behave similarly the specific activity in the available P is equivalent to a specific activity in the isotopically exchanged P and the following equation is fair:

(5) $$\frac{R}{E}=\frac{r}{cp}$$

where R – radioactivity introduced to the sample, Bq.

q – P concentration in tested sample, mg L^−1^
r – radioactivity of the sample, Bq.

### 2.5 Data treatment and statistical analysis

Data sets were tested for normality and homogeneity using Shapiro-Wilk's test and Bartlette's tests respectively. The difference between treatments in each soil variable was subjected to one-way Anova test. The analyses were carried out using GraphPad Prism, version 9.3.

## 3 Results

### 3.1 Soil test P in the amended soils

The distribution of soil test P and Mehlich3 extractable metals in the soils amended with SSP and the recycled fertilisers is shown in Table 4.

**Table 4:** The distribution of Morgan's P, and Mehlich3 P in the P deficient, P balanced, and P saturated soil amended with the recycled DPS products.

P deficient soil				P balanced soil		P saturated soil		
Product	Application rate	Mehlich-3 P, mg kg-1	Morgan's P, mg L-1	Mehlich-3 P, mg kg-1	Morgan's P, mg L-1	Mehlich-3 P, mg kg-1	Morgan's P, mg L-1	
Baseline	N/A	51.8 ± 1.6	2.3 ± 0.1	84 ± 1.7	4.8 ± 0.4	169.7 ± 1.5	12.6 ± 0.1	
SSP	50	86.3 ± 2.4	15.4 ± 0.6	132.8 ± 1.9	15.7 ± 0.5	223.9 ± 3.8	18.7 ± 1.3	
DPS	20	70.0 ± 1.9	6.2 ± 0.2	129.3 ± 0.4	12.3 ± 0.7	208.4 ± 4.4	14.3 ± 0.2	
	50	80.2 ± 2.6	9.3 ± 0.2	147.3 ± 1.2	15.3 ± 1.0	231.0 ± 8.4	17.9 ± 0.3	
	80	113.2 ± 3.9	10.9 ± 1.0	181.2 ± 3.3	16.3 ± 0.6	257.2 ± 6.6	17.5 ± 0.6	
	120	145.1 ± 4.1	17.3 ± 0.4	212.5 ± 4.8	18.9 ± 1.8	304.2 ± 5.6	19.8 ± 0.4	
	150	97.5 ± 1.3	17.4 ± 0.8	172.4 ± 0.4	18.5 ± 2.1	276.7 ± 7.2	21.4 ± 0.8	
	180	156.7 ± 0.9	20.5 ± 2.1	199.1 ± 2.3	22.1 ± 0.3	263.1 ± 3.4	23.0 ± 0.1	
Ash	20	57.9 ± 1.6	2.1 ± 0.1	94.7 ± 1.4	10.5 ± 0.8	186.2 ± 2.5	13.4 ± 0.7	
	50	63.0 ± 1.2	2.7 ± 0.04	102.5 ± 1.3	11.8 ± 0.8	197.5 ± 4.8	14.3 ± 0.7	
	80	75.8 ± 2.8	2.8 ± 0.5	101.9 ± 1.7	12.1 ± 0.8	189.6 ± 2.2	12.9 ± 0.9	
	120	83.7 ± 4.8	3.7 ± 0.8	104.1 ± 6.8	12.3 ± 0.4	219.8 ± 4.4	15.3 ± 0.1	
	150	99.0 ± 2.0	7.0 ± 0.2	115.1 ± 3.9	13.5 ± 0.3	208.9 ± 10.2	14.6 ± 0.3	
	180	117.4 ± 8.8	6.2 ± 0.3	121.8 ± 8.9	14.7 ± 0.3	222.4 ± 14.6	15.4 ± 0.4	
Biochar	20	56.3 ± 1.6	3.3 ± 0.3	101.0 ± 1.5	10.5 ± 0.8	196.3 ± 2.4	12.5 ± 0.3	
	50	76.8 ± 4.7	4.6 ± 1.1	120.1 ± 3.6	12.4 ± 0.1	217.9 ± 1.6	13.1 ± 0.7	
	80	86.4 ± 2.1	8.8 ± 2.2	149.9 ± 5.1	13.3 ± 0.6	228.3 ± 6.6	17.6 ± 1.0	
	120	119.6 ± 2.4	9.1 ± 1.8	185.0 ± 10.6	16.2 ± 0.3	238.8 ± 2.8	17.5 ± 0.3	
	150	135.5 ± 5.4	12.4 ± 0.2	173.8 ± 4.0	16.5 ± 1.4	275.3 ± 8.6	20.4 ± 0.2	
	180	160.4 ± 5.5	13.5 ± 0.6	197.8 ± 12.4	20.4 ± 4.7	278.3 ± 3.6	18.4 ± 2.5	
Hydrochar	20	54.8 ± 0.3	2.3 ± 0.1	96.3 ± 1.7	7.1 ± 0.7	175.4 ± 2.8	8.6 ± 0.2	
	50	38.5 ± 3.7	2.2 ± 0.1	105.4 ± 2.5	8.2 ± 0.1	186.8 ± 2.9	11.7 ± 0.2	
	80	67.5 ± 0.6	2.5 ± 0.8	105.0 ± 1.0	9.6 ± 0.3	190.9 ± 1.7	10.7 ± 1.4	
	120	65.1 ± 1.4	3.3 ± 0.3	111.3 ± 1.6	10.3 ± 0.1	208.8 ± 1.9	12.7 ± 0.5	
	150	107.2 ± 1.6	4.8 ± 0.3	133.8 ± 2.9	12.8 ± 0.5	207.3 ± 2.4	13.5 ± 0.5	
	180	92.8 ± 3.2	4.6 ± 1.8	124.6 ± 2.6	12.4 ± 1.3	201.5 ± 3.6	14.3 ± 0.1	
Struvite	20	53.0 ± 2.3	2.0 ± 0.3	92.3 ± 1.0	12.1 ± 1.1	178.6 ± 2.6	12.4 ± 1.2	
	50	66.2 ± 1.2	7.9 ± 0.6	105.0 ± 1.6	12.2 ± 0.3	198.9 ± 1.6	13.0 ± 0.7	
	80	81.9 ± 0.3	7.8 ± 0.5	119.7 ± 1.5	14.7 ± 1.7	212.1 ± 2.7	13.7 ± 1.2	
	120	103.7 ± 1.2	10.1 ± 0.8	138.3 ± 1.3	15.3 ± 0.8	229.2 ± 1.8	16.5 ± 1.3	
	150	118.6 ± 0.3	12.6 ± 1.3	165.4 ± 6.8	17.1 ± 1.9	269.2 ± 3.8	17.3 ± 0.4	
	180	149.8 ± 1.4	12.4 ± 1.3	181.7 ± 4.5	17.6 ± 0.7	268.2 ± 3.2	18.6 ± 2.1	

**Fig. 1:**
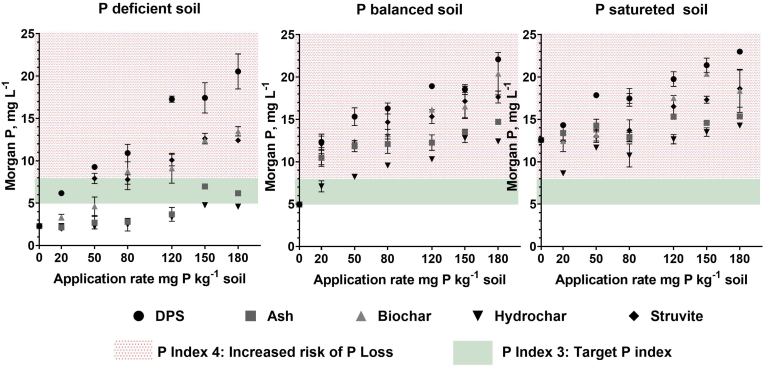
Changes in soil test phosphorus (Morgan's P) with increasing rates of application of dairy processing sludge (DPS) in a P deficient, balanced, and saturated soil. Shaded areas denote soil P Indices for fertiliser advice based on Morgan's P values.

The application of the recycled fertilisers led to an increase in soil test P (Morgan's P and Mehlich3 P), however the magnitude of the increase varied for different fertilisers and initial soil P status (Table 4). The rate of Morgan's P build-up in the P deficient soil was the fastest in soils amended with raw DPS, followed by struvite, biochar, ash, and hydrochar with the slope of Morgan's P increase of 0.098, 0.064, 0.061, 0.026, and 0.015 respectively (Fig. 1). In the P deficient soil, Morgan's P increased from a baseline value of 2.3 mg L ^−1^ to 20.5, 6.2, 13.5, 4.6, and 12.4 mg L ^−1^ when treated with the highest rate (180 mg P kg ^−1^ soil) of DPS, ash, biochar, hydrochar, and struvite, respectively (Table 4). The P deficient soil treated with hydrochar did not reach the target P Index for crop production (P index 3) regardless of application rate, and the maximum Morgan's P recorded in this amendment was 4.8 mg L^−1^. For the other recycled products the following application rates allowed building-up of Morgan's P to the optimal level for crop production: 20 mg P kg ^−1^ soil for DPS, 50 mg P kg ^−1^ soil struvite, 80 mg P kg ^−1^ biochar, and 150 mg P kg ^−1^ ash (Fig. 1 a). A build-up of Morgan's P over 8.0 mg P L^−1^ (P index 4) occurred with some treatments which could pose a risk of P losses into the environment. This transition occurred when the P deficient soil was treated with DPS, biochar, and struvite at application rates of 50 mg P kg ^−1^, 80 mg P kg ^−1^, and 120 mg P kg ^−1^ respectively. Results of the *t*-test showed that no significant differences (p > 0.05) were observed between Morgan's P results for 150 and 180 mg P application rates in ash, hydrochar, and struvite amendment.

In the P balanced soil, the rate of Morgan's P increase was slower compared to the P deficient soil. This increase is expressed as the slope of the linear relationship between Morgan's P and the rate of application. The fastest rate of increase with a slope of 0.074 was recorded in the soil amended with DPS, followed by 0.069 in biochar, 0.056 struvite, 0.038 in ash, and the lowest rate was observed in hydrochar amendments with a slope of 0.036. In this soil, the initial Morgan's P content of 4.9 mg L ^−1^, the upper band of P index 2, transitioned to P index 4 when amended with all the recycled fertilisers (Fig. 1 b).

The P saturated soil also recorded an increase in Morgan's P following applications with recycled fertilisers however the rate of the increase was the slowest compared to the other 2 soils (Fig. 1 c). The highest response was recorded when amended with DPS (slope 0.053) followed by 0.043, 0.037, 0.019, and 0.017 when amended with biochar, struvite, hydrochar, and ash respectively. The highest Morgan's P concentrations recorded in this soil were 23.0, 15.4, 18.4, 14.3, and 18.6 mg P L ^−1^ following application of 180 mg P kg ^−1^ of DPS, ash, biochar, hydrochar, and struvite respectively (Table 4).

The Mehlich3 P reflects both available P and exchangeable P cations with limited availability (Menun et al., 2018). Similarly to changes in the Morgan's P, all the recycled fertilisers increased the Mehlich3 pool (Table 4). Final Mehlich3 P values were the highest in soils amended with DPS, struvite and biochar (Table 4) while thermally treated products, namely ash and hydrochar, were the least effective in increasing Mehlich3 P. The increase in the Mehlich3 P correlated with the application rate of all the recycled fertilisers (p < 0.05).

### 3.2 Rapidly exchangeable P

At the reference application rate of 50 mg P kg^−1^ soil the recycled fertilisers were less effective than chemical fertiliser (SSP) in building up the exchangeable P pool (E value) (Table 5).

**Table 5:** –Rapidly exchangeable P and soil P turnover rates in soils amended with SSP and the recycled fertilisers.

		P deficient soil			P balanced soil			P saturated soil		
Product	Application rate	E value, mg L^−1^	Total P influx, mg P L^−1^ day^−1^	Total P efflux, mg P L^−1^ day^−1^	E value, mg L^−1^	Total P influx, mg P L^−1^ day^−1^	Total P efflux, mg P L^−1^ day^−1^	E value, mg L^−1^	Total P influx, mg P L^−1^ day^−1^	Total P efflux, mg P L^−1^ day^−1^
Baseline	N/A	50.5 ± 3.5	1.6 ± 0.4	3.7 ± 0.6	90.7 ± 7.6	5.0 ± 0.2	5.2 ± 0.4	138.6 ± 16.6	5.5 ± 0.5	6.3 ± 2.1
SSP	50	238.4 ± 24.3	12.8 ± 3.8	11.5 ± 5.2	267.4 ± 4.3	14.1 ± 1.4	15.8 ± 1.2	294.7 ± 32.5	20.9 ± 4.3	20.5 ± 2.5
DPS	20	66.7 ± 10.1	3.8 ± 0.8	3.6 ± 0.6	120.1 ± 10.4	6.1 ± 1.0	9.6 ± 1.7	137.5 ± 22.4	11.8 ± 2.5	13.4 ± 1.1
	50	96.7 ± 7.5	4.0 ± 0.4	5.2 ± 1.4	125.2 ± 3.8	3.8 ± 1.1	8.3 ± 0.9	160.7 ± 28.4	13.6 ± 0.6	13.5 ± 1.0
	80	109.8 ± 2.8	2.3 ± 0.5	4.4 ± 1.1	145.6 ± 4.6	14.3 ± 1.5	15.7 ± 9.7	158.3 ± 9.7	16.9 ± 1.5	17.3 ± 0.8
	120	238.7 ± 48.1	18.4 ± 1.9	14.1 ± 3.3	220.6 ± 19.8	23.0 ± 3.9	19.9 ± 6.5	144.4 ± 5.8	30.8 ± 1.4	26.2 ± 1.0
	150	244.4 ± 6.5	18.3 ± 2.7	16.3 ± 4.8	293.2 ± 17.2	21.8 ± 2.8	21.6 ± 3.0	319.9 ± 16.2	17.5 ± 0.1	14.5 ± 1.1
	180	245.6 ± 6.09	21.7 ± 2.9	19.4 ± 0.6	278.7 ± 27.8	26.4 ± 2.9	21.5 ± 2.4	294.5 ± 7.2	19.9 ± 2.2	15.8 ± 2.4
Ash	20	19.3 ± 0.5	1.4 ± 0.1	2.6 ± 0.1	68.9 ± 3.2	1.5 ± 1.3	2.4 ± 1.3	91.0 ± 3.0	1.1 ± 0.1	3.2 ± 1.2
	50	28.7 ± 3.5	0.7 ± 0.1	1.0 ± 0.5	75.1 ± 9.1	1.6 ± 1.8	2.3 ± 0.7	153.0 ± 13.9	9.4 ± 1.8	9.2 ± 1.3
	80	60.1 ± 13.7	1.8 ± 1.5	2.5 ± 1.0	120.1 ± 6.2	7.9 ± 0.3	7.9 ± 1.2	154.9 ± 8.8	5.2 ± 2.4	5.3 ± 0.9
	120	31.8 ± 6.3	3.3 ± 0.7	5.7 ± 0.9	82.8 ± 0.5	2.2 ± 0.7	3.5 ± 0.9	94.9 ± 8.1	5.0 ± 0.5	6.0 ± 0.4
	150	57.0 ± 6.4	2.8 ± 0.4	4.4 ± 0.8	92.0 ± 7.1	1.9 ± 0.8	2.8 ± 2.3	131.8 ± 8.8	2.7 ± 0.7	2.2 ± 08
	180	81.2 ± 1.0	1.7 ± 0.1	2.0 ± 0.2	149.7 ± 9.5	9.6 ± 2.2	8.2 ± 1.9	137.4 ± 14.2	1.8 ± 1.1	1.2 ± 0.3
Biochar	20	33.0 ± 3.2	4.4 ± 0.7	9.2 ± 1.04	70.1 ± 11.2	3.0 ± 1.7	9.6 ± 3.2	83.4 ± 7.3	2.8 ± 1.3	9.8 ± 1.5
	50	45.4 ± 7.3	3.1 ± 0.8	8.1 ± 1.3	67.5 ± 5.5	1.6 ± 0.9	7.0 ± 0.9	84.0 ± 12.7	1.6 ± 0.9	8.3 ± 1.1
	80	59.2 ± 12.8	2.3 ± 1.0	8.0 ± 1.0	71.1 ± 3.4	2.2 ± 0.9	7.9 ± 0.5	97.7 ± 4.2	2.1 ± 1.2	6.8 ± 0.2
	120	65.4 ± 6.9	3.8 ± 1.3	13.2 ± 2.1	79.5 ± 2.9	1.1 ± 0.7	4.2 ± 0.9	109.9 ± 3.0	1.0 ± 0.7	6.3 ± 0.3
	150	86.7 ± 0.1	2.6 ± 0.6	9.7 ± 1.0	88.4 ± 0.7	2.2 ± 1.4	9.1 ± 1.0	113.6 ± 5.1	2.9 ± 0.7	10.6 ± 1.4
	180	78.4 ± 5.5	2.5 ± 1.4	10.4 ± 1.5	92.7 ± 13.7	2.7 ± 0.9	9.7 ± 1.7	110.1 ± 10.7	1.3 ± 0.7	8.8 ± 1.8
Hydrochar	20	19.8 ± 0.7	1.3 ± 0.2	2.5 ± 0.2	61.8 ± 5.2	3.6 ± 0.9	7.4 ± 1.3	104.2 ± 4.8	1.0 ± 0.7	4.5 ± 0.6
	50	22.8 ± 1.6	0.8 ± 0.1	1.6 ± 0.1	75.7 ± 1.7	1.8 ± 0.1	4.4 ± 0.03	118.8 ± 20.9	1.5 ± 0.8	2.1 ± 0.9
	80	22.7 ± 2.9	1.2 ± 0.5	1.9 ± 1.4	65.2 ± 0.9	1.9 ± 0.9	2.8 ± 1.1	102.3 ± 13.6	1.8 ± 0.2	2.4 ± 1.6
	120	30.2 ± 3.2	1.0 ± 0.6	2.2 ± 1.1	73.9 ± 0.9	0.9 ± 0.2	1.5 ± 0.5	110.9 ± 5.0	0.7 ± 0.2	0.6 ± 0.3
	150	35.5 ± 0.4	0.3 ± 0.1	0.9 ± 0.4	69.4 ± 3.9	0.3 ± 0.1	2.2 ± 0.7	93.9 ± 0.9	0.9 ± 0.1	2.6 ± 0.8
	180	36.1 ± 1.8	1.0 ± 0.4	2.1 ± 0.2	92.8 ± 8.6	0.7 ± 0.4	2.2 ± 0.3	107.7 ± 1.8	0.4 ± 0.2	2.4 ± 0.3
Struvite	20	94.2 ± 14.6	3.4 ± 1.7	5.3 ± 1.5	151.7 ± 33.6	12.4 ± 1.7	12.1 ± 2.0	137.7 ± 21.6	9.2 ± 2.5	9.9 ± 1.3
	50	89.6 ± 12.7	5.5 ± 1.2	5.1 ± 1.0	107.6 ± 20.5	9.0 ± 3.9	8.8 ± 3.6	136.7 ± 27.8	9.3 ± 2.8	13.8 ± 3.2
	80	91.8 ± 10.1	6.3 ± 1.3	7.7 ± 2.9	123.5 ± 12.9	10.9 ± 2.3	11.5 ± 1.3	177.7 ± 19.6	16.5 ± 4.3	20.7 ± 3.1
	120	114.3 ± 11.7	9.1 ± 2.0	10.2 ± 1.6	124.2 ± 11.2	12.1 ± 2.5	12.0 ± 1.9	177.3 ± 13.3	14.1 ± 2.8	18.3 ± 1.8
	150	120.9 ± 16.1	9.3 ± 2.4	8.3 ± 1.4	134.8 ± 0.6	11.9 ± 1.6	12.1 ± 0.8	155.3 ± 7.0	11.7 ± 2.9	15.0 ± 2.7
	180	135.0 ± 11.0	11.3 ± 1.7	12.0 ± 1.6	141.4 ± 7.8	13.9 ± 1.9	16.0 ± 1.2	186.5 ± 10.7	17.1 ± 1.3	20.1 ± 2.6

**Fig. 2:**
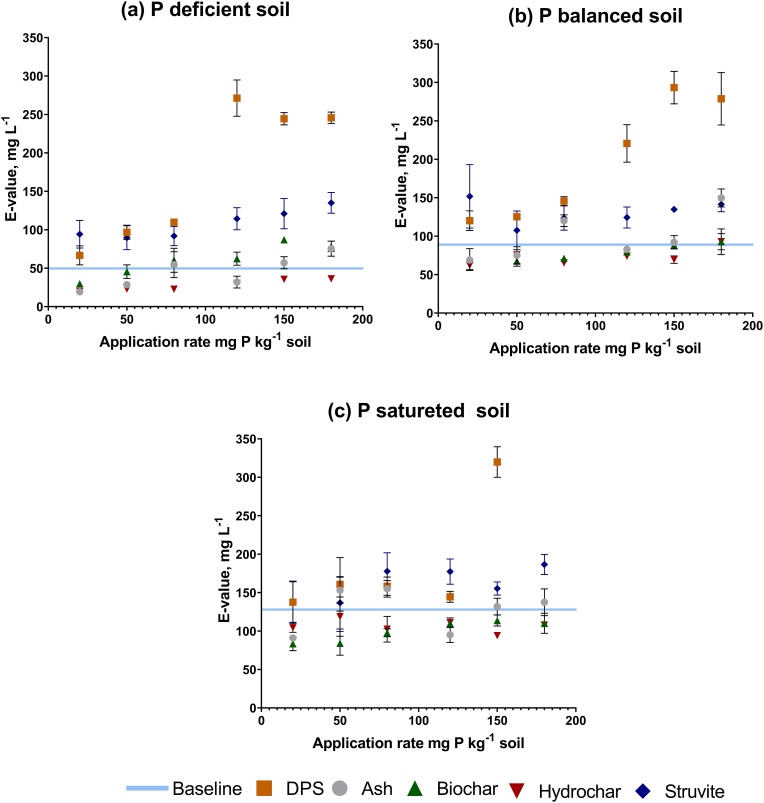
Changes in rapidly exchangeable P (E value) in soils amended with recycled fertilisers. The blue line indicates the baseline E value in unamended soils that were P deficient, balanced and saturated. (For interpretation of the references to colour in this figure legend, the reader is referred to the Web version of this article.)

In the P deficient soil the application of ash, biochar, and hydrochar at 20 mg P kg^−1^ soil led to a decrease in the rapidly exchangeable P by 62%, 35%, and 61% below initial baseline values of 50.5 mg L^−1^. This is demonstrated on Fig. 2a where the blue line shows the baseline E value in this soil which did not receive any fertiliser under long-term P management. Increasing the application rates of ash and biochar allowed a build-up of the rapidly exchangeable P pool. At the application rate of 80 mg P kg^−1^ ash and biochar E value recovered to above baseline values (Table 5). The E values fell below the baseline values following the application of hydrochar and did not recover, regardless of the application rate. Unlike the thermally treated products, the DPS and struvite products, even at low application rates, led to an increase in the rapidly exchangeable P pool. The DPS amendment resulted in a gradual increase in E values initially, followed by a sharp increase at high application rates of DPS (120 mg P kg^−1^ soil and above). The application of DPS at the rate of 120 mg P led to a two-fold increase in E relative to the 80 mg P kg^−1^ soil rate (Table 5). In the DPS amendment the E value peaked in the soil which received the highest dose of DPS (180 mg P) (Table 5). The rate of increase in E value following the application of struvite was more gradual with the highest recorded value of 135 mg L^−1^, an almost threefold increase from the baseline (Table 5).

Similarly, in the P balanced soil the application of the thermally treated products at the lowest application rate of 20 mg P led to a decrease in E from the baseline value of 90.7 mg L^−1^. The application of ash, biochar, and hydrochar resulted in a decrease in the E value of 25%, 22, and 32% respectively below the baseline value (Fig. 2b). The E value was recovered to baseline following application rates of 80 mg P for ash amendment and 180 mg P for biochar and hydrochar amendments. Similar to the P deficient soil, the application of the raw DPS and struvite increased the E value even at the lowest application rates. An increase in the E value was gradual with the struvite amendment with the highest value of 141 mg L^−1^. In the DPS amendment the highest recorded E value was 278.7 mg L^−1^ (Fig. 2).

The P saturated soil exhibited the slowest rate of the exchangeable P pool build-up compared to the other soils. The thermally treated products led to a decrease in the E below the baseline value when applied at the low application rate. The DPS and struvite did not alter the E value at the application rate of 20 mg P (Fig. 2c) and no significant increase in E value was recorded until the application rate reached 50 mg L^−1^ (Fig. 2c). The application of 20 mg P of ash, biochar, and hydrochar led to a decrease in E value by 31%, 40%, and 25% respectively, similar to that observed in the deficient and balanced soil. In the biochar and hydrochar amend soils, the application of these products at the higher application rates did not recover the E value to the baseline value. In the ash amended P saturated soil, the E value fluctuated with the increasing application rate and no correlation between the ash application rate and E value was observed. At the highest application rate of struvite and DPS, the E value increased up to 186.5 and 294.5 mg L^−1^ respectively.

### 3.3 Soil P turnover rates

The soil P turnover rates measured in the present study reflect the daily P flux from the available P pool into the exchangeable P pool (Total P efflux) and the reverse flux reflecting the daily P transfer from the exchangeable P pool into the available P pool (Total P influx). In the P deficient soil the balance in the P turnover rates shifted towards P influx at the application rate of 120 mg P kg^−1^ soil and was associated with a sharp increase in E value (Table 5). At the highest application rate of DPS (180 mg P kg^−1^) the total P influx was thirteen times higher compared to the baseline value of 1.6 ± 0.4 mg P L^−1^ day^−1^ and the total P efflux increased fivefold from 3.7 ± 0.6 mg P L^−1^ day^−1^ to 19.4 mg P L^−1^ day^−1^. Similarly, the application of struvite led to an increase in the P turnover rates which correlated with the application rate.). Unlike in the DPS amendment, P fluxes were more balanced across all application rates of struvite, and equilibrium was achieved between the P fluxes in the soil following application of 120 mg P kg^−1^ soil struvite. The total daily P turnover rates were lower compared to the rates in the soil amended with DPS with a sevenfold increase in the total P influx and a threefold increase in the total P efflux at the highest application rate of 180 mg P resulting in the daily total influx of 11.3 and 12.0 mg P L^−1^ day^−1^ (Table 5). Among the three thermally treated products biochar amended P deficient soil recorded the highest rates of increase in total P flux. The total P influx in the P deficient soil treated with biochar at the highest application rate of 180 mg P kg^−1^ soil increased by 56% and the total P efflux increased threefold from the baseline values (Table 5). The distribution of the total P fluxes remained unbalanced with flux predominantly in the direction of total P efflux. In the P deficient soil amended with ash and hydrochar changes in flux were inconsistent and no significant correlation between the product application rate and total P fluxes was observed (P > 0.05). The total P fluxes in the P deficient soil decreased (Table 5) following the application of hydrochar. The P fluxes remained unbalanced with the total P efflux being the predominant P flux.

In the P balanced soil the application of DPS increased total P turnover rates from the baseline value (Table 5). At the highest application rate of 180 mg P the total influx was fivefold higher and the total P efflux was fourfold higher compared to the baseline values of 5.0 ± 0.2 mg P L^−1^ day^−1^ and 5.2 ± 0.1 mg P L^−1^ day^−1^ respectively. At the lower application rates (20–80 mg P kg^−1^ soil) the preferred mechanism was a build-up of the rapidly exchangeable P as the predominant P efflux was the total P efflux. As application rates increased (120–180 mg P kg^−1^ soil) the P turnover shifted towards total P influx becoming the predominant P flux (Table 5). The application of struvite led to a threefold increase in both total P influx and total P efflux (Table 5) which remained balanced regardless of the product application rate. In the biochar amendment a decrease in the total P influx from baseline values was observed (Table 5). At the highest application rate total P influx was twofold lower compared to the baseline. The total P efflux increased twofold in the biochar amended soils resulting in a shifting flux distribution towards the total P efflux. The changes in the total P efflux were associated with the application of biochar but no significant correlation between the application rate and total P fluxes was observed. Similarly to the P deficient soil, the application of hydrochar and ash led to a decrease in the total P fluxes (Table 5) compared to the baseline values. No correlation between the product's application rate and total P fluxes was observed and only applications at 80 and 180 mg P increased P flux above baseline values. A threefold and twofold decrease in total influx and efflux respectively was observed when ash was added at rates of 20, 50, 120 and 150 mg P kg^−1^ soil. A shift of the total P turnover rates from balanced to a predominant P efflux was recorded in the P balanced soils amended with ash. A tenfold decrease in the total P influx and a twofold decrease in the total P efflux was recorded in the P balanced soil following the application of 180 mg P hydrochar. At all application rates application of the hydrochar changed the P turnover rates distribution with the predominant total P efflux (Table 5).

Similar trends were observed in the P saturated soil. The application of the DPS and struvite increased the total P turnover rates from the baseline values of 5.5 ± 0.5 mg P L^−1^ day^−1^ total P influx and 6.3 ± 2.1 mg P L^−1^ day^−1^ total P efflux. At the highest application rate of DPS the total P influx increased fourfold from the baseline and the total P efflux increased almost threefold resulting in total P turnover with total P influx being a preferred mechanism in soil P turnover. The shift in P turnover balance occurred at the application rate of 120 mg P DPS (Table 5) in this soil. The total P influx and total P efflux in the P saturated soil treated with 180 mg P were 17.1 and 21.2 mg P L^−1^ day ^−1^. The relatively balanced distribution of the total P fluxes was reported in the unamended P saturated soil was sustained in the samples treated with 20 mg P struvite, however, this balance shifted towards dominating total P efflux following application >50 mg P struvite. Similarly to the trends observed in the P balanced soil, the biochar and hydrochar amendments led to an increase in the total P efflux and a decrease in the total P influx (Table 5). No correlation between the total P fluxes and application rate was observed. The resulting total P fluxes in the amended P saturated soil were unbalanced with the P efflux being the predominant value. In the ash amended P saturated soil no correlation between the total P flux and application rate was observed. At the application rate of 80 and 120 mg P the P turnover rates were comparable to the baseline values and balanced. The soil P turnover rates in the P saturated soil amended with 20, 150, and 180 mg P kg^−1^ soil of ash (Table 5) were slower than baseline values resulting in a decline in turnover rates following ash application.

## 4 Discussion

### 4.1 Building up the available phosphorus in P deficient soils using recycled fertilisers

With the exception of hydrochar, all of the recycled fertiliser product enabled build up of the available P pool in the P deficient soil to the target P level for crop production (5.0 mg P L^−1^ soil). The resulting effectiveness of the recycled fertilisers for Morgan's P build-up relative to SSP (being 100% effective) ranged from 1% in the hydrochar to 44.7% using DPS. The most effective recycled fertilisers were raw DPS and struvite. The optimal rates of the recycled fertilisers which allowed build up the available P level in the P deficient soil were 20 mg P kg^−1^ DPS and struvite, 50 mg P kg^−1^ biochar, 150 mg P kg^−1^ soil ash and over 180 mg P kg^−1^ soil hydrochar. Some earlier studies reported that the fertiliser equivalency value (FEV) of the recycled products, and DPS in particular, are lower compared to mineral fertilisers immediately after application, and increase with time (Ashekuzzaman et al., 2021a, Ashekuzzaman et al., 2021b; Ashekuzzaman et al., 2021b). Ashekuzzaman et al. (2021a) reported that FEV for alum-treated DPS, on the first harvest was 50% and increased to 109% cumulatively over 4 harvests) using DPS of similar composition and origin to the DSP used in the present study. This could be due to a lower P solubility in the recycled fertilisers (Vanden et al., 2021) with a larger fraction of P added with the recycled products being transferred to a labile P pool. While the availability is limited immediately after application a fraction of the labile P from the recycled fertilisers becomes available for crops over the growing season Khomenko et al. (submitted). The potential of the recycled fertilisers to release available P into the available P pool and their impact on P dynamics are discussed in section 4.3.

Current recommendations for soil P build-up on P deficient soils and P draw-down on P saturated soils are designed around managing conventional fertilisers with a high fraction of soluble P. Moreover, existing Programmes of Measures (POMs) S.I. No. 605 of 2017 (European Union, 2017) for agriculture under the EU Water Framework Directive (WFD) (2000/60/IEC) and Nitrates Directive (91/676/EEC) requiring saturated P soils to decline down to environmentally sustainable P levels are time bound and based on mineral P which have faster P turnover in soils. These timeframes (for build-up and decline) need to be updated to account for mechanistic behaviours of the recycled fertilisers allowing for longer P release and draw-down in soils. As acknowledged by the current EU Fertilising Products Regulation (EC, 2019) some raw wastes and their derived secondary products have shown promise as bio-based alternatives in recent years. However, the potential of these alternatives is largely based on a subset of wastes that have been tested extensively (e.g. human biosolids). As new wastes emerge within the bioeconomy there has been an over reliance on assuming all wastes offer the same potential based on nutrient profiles. Recent studies on DPS have shown that nutrient and metal profiles can be somewhat misleading when it comes to actual agronomic performance Khomenko et al. (submitted) Ashekuzzaman et al., 2021a, Ashekuzzaman et al., 2021b; Shi et al., 2022), and these differences need to be reflected in the updated legislation.

### 4.2 Phosphorus availability in soils amended with recycled fertilisers as a function of product type

Among all the recycled products used in this study, DPS, struvite, and biochar showed the greatest potential to increase soil test P. However the resulting soil test P values at the reference application rate were lower compared to the SSP amendment and higher application rates for these products were required to reach the target P index. Lower P availability was observed in the soil amended with the recycled fertilisers compared to mineral fertilisers and can be associated with the composition and method of the waste recycling. Previous research reported that the presence of metal-bound phosphorus in recycled sewage sludge (Nanzer et al., 2014; Wollmann et al., 2018) and other bio-based fertilisers (Frossard et al., 2002; Komiyama and Ito, 2019) were associated with lower P availability. Due to the chemical precipitation of P at wastewater treatment facilities DPS sludge contains a high amount of multivalent metals (Shi et al., 2021) potentially impacting P solubility in the recycled products. The products used in the present study were treated using different P precipitating agents and products with high Aluminium (DPS and biochar), Calcium (ash), and Iron (hydrochar) content were tested (Table 3). The alum-treated products tested in the present study were more effective in building up the available P pool compared to Ca- and Fe-treated products. The presence of Al in recycled fertilisers was previously reported to be associated with higher P availability owing to binding of P on Al (hydr)oxides which prevent the formation of less soluble Ca phosphates (Vanden et al., 2021) thereby improving the availability of P in the products.

The least effective products, as evidenced from the slowest P build-up rates, were Ca-treated ash (pH 9.33) and Fe-treated hydrochar. Earlier studies on P speciation and extractability from sewage sludge treated with Ca reported that a large fraction of P in such products is represented as tribasic phosphates insoluble in water (Daneshgar et al., 2018; Vogel et al., 2011). This was also confirmed by Nanzer et al. (2014) who found that the larger fraction of P from thermo-chemically treated sewage sludge ash applied to soils was incorporated into the HCl-extractable P pool which is associated with non-available Ca-bound P. Similarly, high Fe content in bio-based fertilisers was reported to be associated with limited P availability and P use efficiency in soils amended with bio-based fertilisers. This effect is likely associated with the ability of Fe (hydr)oxides to adsorb phosphates preventing the formation of available P forms (Vanden et al., 2021).

The impact of processing steps on P availability in bio-based fertilisers was previously reported by Christel et al. (2014), Nanzer et al. (2014), and Vandecasteele et al. (2017). The authors suggest that raw material has the highest P availability which decreases with drying, composting, pyrolysis, and combustion. Thermal treatment of waste alters crystalline phases in the final products increasing the fraction of hydroxyl-apatite P-forms (Peplinski et al., 2009; Nanzer et al., 2014) with higher processing temperatures typically associated with lower P availability (Gul and Whalen, 2016). Some of the thermally treated products tested in the present study followed this pattern with ash having lower P availability than biochar, struvite, and raw DPS. However hydrochar, which is produced at the lowest temperature (180–260 °C) (Ghanim et al., 2018), demonstrated the lowest efficiency in available P build-up. Available studies report that besides processing temperature, other parameters, such as pH, residence time, and the presence of multivalent metals impact nutrient solubility in hydrochars (Ekpo et al., 2016; Ghanim et al., 2018). The presence of Al, Ca, Mg and Fe have been widely reported to contribute to the formation of insoluble P forms in hydrochar produced from various streams (Dai et al., 2015; Heilmann et al., 2014; Qian and Jiang, 2014). This indicates that the P availability of recycled products is a function of both chemical composition and processing parameters.

### 4.3 Isotopically exchangeable P in amended soils: impacts on P turnover and rapidly exchangeable P

**Fig. 3:**
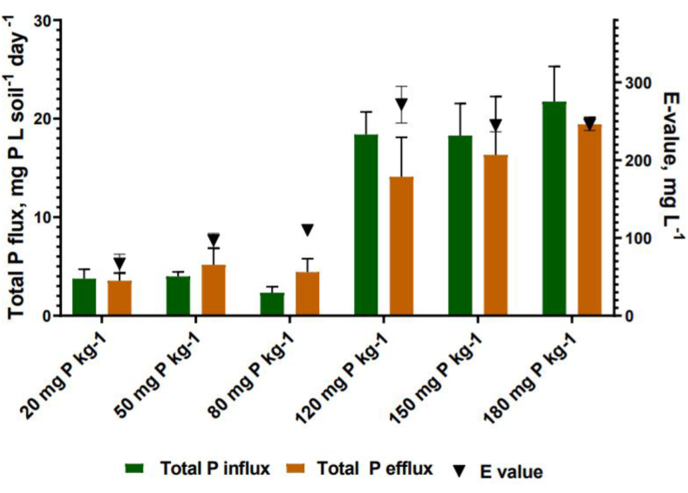
The shift in the distribution of total P flux between influx and efflux in the P deficient soil treated with dairy processing sludge (DPS). Graph shows the shift from the predominant total P efflux to total P influx and the step change in E values.

In the present study changes in the P flux from predominantly P efflux (in P deficient soil) and balanced P fluxes (in P saturated soil) to a predominant total P influx only occurred in the soils amended with raw DPS and coincided with a step change in E value (Fig. 3). The rate of P influx increase was faster compared to the P efflux increase rate when P deficient soil is amended with DPS. The higher response observed in the total P influx is likely associated with an injection of a large fraction of soluble phosphates which are released from the DPS increasing the abiotic P influx into the available P pools which is reflected in a higher total P influx. The struvite amendment led to an increase in both E value and exchangeable P pool without a shift in the direction of soil P flux (Fig. 3). A decrease in both P turnover rates and E values were recorded in soil treated with thermally treated products (Fig. 3). An increase in the P turnover rates and exchangeable P pool in the soils amended with DPS and struvite was caused by an increase of the available P in the soil following application of fertilisers with a high fraction of soluble P. Wanek et al. (2019) and Achat et al. (2016) reported that physiochemical P cycling in the soils strongly correlated with the available P regardless of the soils pH, total P content and texture. When the soil was treated with the DPS at the lower application rates (20–80 mg P kg^−1^ soil) the total P efflux was higher compared to the total P influx suggesting that in these amendments building up the exchangeable P pool was a predominant mechanism. At the application rate of 120 mg P kg^−1^ soil the balance between the total P influx and total P efflux has shifted (Fig. 3). After a shift in the total P flux distribution the increase in the rapidly exchangeable P pool slowed down (Fig. 2) and the final E values were comparable suggesting that the exchangeable P pool was saturated. The application of struvite did not allow a build-up of E to the same threshold level, probably, due to lower solubility and slower P release from the struvite.

The opposite effect was observed when the thermally treated products were applied to soils. An increased total P efflux and gradual increase in the E value suggests that application of the thermally treated products resulted in the immobilisation of the available P following application of these fertilisers across all soil P indices. In the present study an acidic soil was tested and the application of the products high in Al3+ and Fe2+/Fe3+ could potentially lead to immobilisation of P due to the formation of Al-and Fe-phosphates (Glaser and Lehr, 2019). Some studies reported that biochar has a high affinity for P and can potentially reduce P availability in soils (Rashmi and Biswas, 2020). A similar effect was reported by Louise et al. (2018) who found that biochars produced at higher temperatures had high P sorption capacity and affinity to P. In the present study a decrease in the total P influx following application of the biochar and a predominant P efflux into the exchangeable P pool was recorded across all soil indices indicated that P sorption was a predominant P flux in acidic soils amended with biochar. An increase in the total P efflux in the soils amended with biochar suggests that P is transferred into the rapidly exchangeable P pool.

Hydrochars generally have reduced P solubility and reactivity in the solid phase due to an elevated fraction of apatite P (Möller et al., 2018). In the present study the low availability of P in hydrochar was reflected in its poor ability to increase both soil test P and exchangeable P pools across all soil indices. Similarly to the effect of biochars, high affinity for P and low P release from hydrochar was previously reported in the literature (Takaya et al., 2016). In the present study a decrease in the rapidly exchangeable P pool and P turnover rates were recorded following the application of hydrochar across all P indices indicating an immobilisation of P in non-exchangeable soil P reserves with decreased soil P adsorption/desorption processes as evidenced by decreased P turnover rates (Wanek et al., 2019).

An elevated concentration of Ca and high pH of ash can create additional P binding sites (Günther et al., 2018) promoting available P immobilisation following the application of ash. A predominant P efflux recorded in soils amended with thermally treated products suggests that P sorption was a predominant mechanism in these amendments. However the overall lower P turnover rates compared to baseline suggest that a fraction of P immobilized in thermally treated products suggest that a fraction of immobilized P could be transferred to a pool which is not sensitive to the IPD assay. The available and rapidly exchangeable P pools and daily P turnover rates were measured in the present study over 24 h period using Morgan's P extracting solution. Using a stronger extracting solution or increasing the time of the IPD assay could provide more information on P turnover in slowly exchangeable P pools following the application of these products. However previous studies reported that while a strong P extracting solution can provide means to estimate the size of the slowly exchangeable P pool fractions, it cannot be used as an indicator of P availability (Six et al., 2012) and is associated with legacy P and non-available P fractions.

Deployment of the isotopic tools can aid in extending our understanding of how bio-based fertilisers impact mechanisms of available soil P turnover and build-up. Understanding differences in mechanistic behaviour offers insights into the potential agronomic performance of novel recycled P fertilisers and suggest optimal application rates to maximise P availability to match crop demand. Such techniques unlock knowledge gaps relating to P availability and rates of P release in soils amended with recycled fertilisers and offer greater explanatory power with respect to their expected agronomic performance. In the present study IPD technique was applied at the bench scale to soil incubated with novel fertiliser products, to examine aspects of the soil P cycle without considering the soil-plant interaction. The findings described here offer valuable information regarding optimal application rates for P build-up and highlight differences in soil P build-up rates between fertilisers produced using thermal and chemical processes. However, future research could include crops to describe P uptake and contribution of P dissolution to P release from the recycled fertilisers. Isotope studies under growing conditions will enable incorporation of wastes into on-farm nutrient management planning (including safe application rates based on soil metal contents) and enable a just transition from fossil based fertiliser to bio-based wastes.

## 5 Conclusions

All of the recycled fertilisers were less effective in building up available and exchangeable P pools compared to the mineral fertilisers and had a varying effect on the P turnover dynamics. The highest P availability was recorded in the soils amended with the raw alum-treated DPS and the DPS-derived struvite. Thermally treated fertilisers demonstrated poor performance in building up the available P pool with the Fe-rich hydrochar and Ca-rich ash being the least effective products for available P build-up. A high concentration of multivalent metals in the initial waste stream and thermal treatment of the waste limits P availability even when the processing involves moderate temperatures.

The application of raw DPS and struvite led to an increase in P turnover rates and a build-up of the exchangeable P pool across all P indices. In the P deficient soil with the predominant total P efflux the application of these products enabled to reach a more balanced total P flux distribution (struvite amendment) or shifted the distribution of the P flux towards the predominant total P influx which is associated with a saturation of the exchangeable P pool. Thermally treated products had the opposite effect on the soil P dynamics causing a decrease in soil P turnover rates with the P efflux becoming a predominant P flux even in the P balanced and P saturated soils. The changes in the P turnover rates were also reflected in decrease in the available and exchangeable P pools below the baseline values following the application of low doses of the thermally treated products.

Raw waste and products produced without thermal processing have a high potential to replace mineral P fertilisers and build up available P in P deficient soils. However slower P turnover rates in soils amended with the recycled products compared to mineral fertilisers indicate that application rates need to be increased or application time needs to be adjusted in order to allow sufficient time for a build-up of the available and exchangeable P pools. Thermally treated products with high concentration of multivalent metals are not effective in building up the available P to a target level in the P deficient soils and may lead to immobilisation of the available P at low application rate further restricting P availability for crops.

## Author contributions

**O.K.**: Conceptualization, Methodology, Data curation, Writing – original draft. **K.D.** –Conceptualization, Methodology, Writing – review & editing, Supervision, Funding acquisition. **O.F.** – Conceptualization, Writing – review & editing, Supervision. **J.J.L.** – Funding acquisition.

## Declaration of competing interest

The authors declare that they have no known competing financial interests or personal relationships that could have appeared to influence the work reported in this paper.

## Data Availability

The data that has been used is confidential.
